# Emergence of *Haemophilus influenzae* Strains in the Nasopharynx of Children with Tuberculosis

**DOI:** 10.1155/2011/420284

**Published:** 2011-07-03

**Authors:** Alina V. Martynova, Larisa A. Balabanova, Alexei Pruschinskyi

**Affiliations:** ^1^Epidemiology Department, State Vladivostok Medical University, Ostryakova 2, Vladivostok, Russia; ^2^Pacific Institute of Bioorganic Chemistry (PIBOC), Russia

## Abstract

Being rigorously studied, epidemiology aspects of *Haemophilus influenzae* carriage are still remaining unclear. Especially it concerns such a group as children with low immune status and also such group as children with tuberculosis infection. We examined nasopharyngeal tract of children with tuberculosis infection in remission and checked how often these children are carriers of *Haemophilus influenzae* strains. Also we gained microbiology characteristics of the isolates and defined the clinical significance of *H. influenzae* carriage in development of opportunistic infections in children with the tuberculosis infection.

## 1. Introduction

Despite improvements in diagnosis, treatment,and preventive measures, tuberculosis remains an important cause of morbidity globally. 

In the Primorye Region of far east Russia (population 2000.000), the incidence of tuberculosis in 2008 was 105.7 per 100.000 and with a prevalence of 222 per 100.000. For children under 17 years, the incidence of tuberculosis was 36.6 per 100.000. In 2008, there were 108 cases of tuberculosis in  children under 11 years, and 83 cases in children from 11 to 17 years (Federal Morbidity Report, 2009). Mortality increased by 20.7% compared to 2000. In the pediatric population, pulmonary tuberculosis accounted for 90.9% of all tuberculosis in 2008, up from 72.7% in 1999.

It was recognized that children treated for tuberculosis were more often ill with other bacterial respiratory tract infections than children without such comorbidity. There are several possible contributing factors to this, for example, impaired immune status, malnutrition, and low  socioeconomical status of their family. However, other important factors could be the regular antimicrobial (antituberculosis) chemotherapy they receive or attendance at the Pediatric Tuberculosis Center for anything between 20 and 180 days, according to the current tuberculosis treatment protocol. As the main focus for such patients is the treatment of tuberculosis, the problem of other bacterial respiratory tract infections could be underestimated. 

There are some data on the carriage of bacterial respiratory tract pathogens in children with various comorbidities [1–5], but little information on the carriage of *H.influenzae* in children with tuberculosis or children from closed communities [5, 6].

**Aim**

We aim to study the prevalance of *H. influenzae *in the  nasopharynx of children with tuberculosis and to assess antimicrobial susceptibility of these bacteria.

## 2. Materials and Methods

We examined 85 children from the Pediatric Tuberculosis Center (Group 1), all of whom had taken antituberculosis chemotherapy within the previous 1.5-2  months, with rifampicin, isoniazid, ethambutol, and streptomycin (according to WHO recommendations). They had received their treatment in the Pediatris Tuberculosis Center sanatorium for up to 3 months. Of the 85 patients in Group 1, 36 patients (42.3%) had tuberculosis intoxication, 28 patients (32.9%) had post-BCG complications, and 21 patients (24.7%) had extrapulmonary tuberculosis. Group 1 consisted of 41 patients (48.2%) under 5 years of age, 28 patients (32.94%) aged 6–10 years, and 16 patients (18.8%) aged 11–15 years. All Group 1 patients took low doses of rifampicin as recommended by the Public Health Ministry. A control group (Group 2) consisted of age-matched children without tuberculosis attending the pediatric sanatorium with gastrointestinal pathology (70 patients).

Nasal- and oropharyngeal swabs were taken from both groups of children using nylon flocked  swabs and a liquid transport medium, which were plated onto an appropriate medium (Chocolate agar, Biorad, USA). 

The identification of *H. influenzae *strains was confirmed by the use of X factor (hemin), V factor (nicotinamide-adenine dinucleotide), and X+V factor disks (Lachema, India) on  nutrient agar plates according to the manufacturer's instructions. Serotypes were determined by means of a coagglutination technique with antiserum against serotype bcapsular polysaccharide and polyvalent antiserum for serotypes a, c, d, e, and f (Phadebact Hitypes a, c–f, Boule Diagnostics, Sweden). The following six strains were used as positive controls for  serotypes a, b, c, d, e and f, respectively : ATCC 9006, ATCC 10211, ATCC 9007, ATCC 9332, ATTC 8142 and ATCC 9833. Initial typing results were confirmed by typing all strains by PCR by the method of Maaroufi [7]. The presence of *β*-lactamase was detected with  nitrocefin disks (BBL Cefinase, Becton Dickinson, Cockeysville, Md, USA). Antimicrobial susceptibility was determined by disk diffusion and microdilution methods according to CLSI (Clinical and Laboratory Standards Institute) standards and breakpoints. For macrolides (clarithromycin), resistance was taken as  MIC ≥ 32 mg/L, for rifampicin, it was about ≥ 4 mg/L, and for ampicillin it was ≥ 4 mg/L. The study was approved by the Local Human Investigations Committee. Data were analysed according to common statistical methods, including incidence and prevalence analysis and odds ratio (OR) calculating for risk factors analysis.

## 3. Results

More than 60% of the children with tuberculosis had acute pathology of upper and lower respiratory tract of nontuberculosis etiology more than 8 times per year. Children younger of 5 years with tuberculosis had upper respiratory tract pathology more often (OR = 9.0) than children of the same age without tuberculosis. In children without  tuberculosis, there were only 8 who had acute pathology of upper and lower respiratory tract more than 5 times per year).

A total of 51 strains of *H.influenzae *were isolated from 43 patients in Group 1, and 23 strains from 18 patients in the control group (Group 2). The results are presented in Table 1.

Children from Group 1 carried *H. influenzae *more often (50.5%, 43 patients) than children from Group 2 (25.5% 18 patients). It is notable, however, that antimicrobial consumption was much higher in children of the 1st Group (*P* =  0.003). Children in Group 1 often harbored more than 1 strain of *H. influenzae*. 

From Group 1 children, there were 51 strains isolated from 43 patients. Only one strain in 26 patients (60.4%), and 2 and more strains in 17 patients (39.5%). From Group 2 children, there was a single strain from13 patients (18.57%) and 2 or more strains from 5 patients (7,1%) (*P* <  0.05).

The prevalence of different serotypes of Haemophilus genus was also different in children from Group 1, compared to children in Group 2. In Group 1, 54% (28 strains) were nontypable, and 46% (23 strains) were typable  *H. influenzae*. In Group 2, 24,8% (17 strains) were typable, and only 8.57% (6 strains) were nontypable strains of *H. influenzae.* In Group 1 the types were type B 47.82 % (11  isolates), type A  13.04 % (3 strains), type F 17.31% (4 strains), type D 8.69% (2 strains), and type C 13.04% (3strains). In Group 2, the types were 15,7% (11isolates) of type B, 2.85% (2 isolates) of type D, 2.85% (2 isolates) of type C, and 2.85% (on 1  isolate) of types E and F.

Regarding beta-lactamase production, there were 12 beta-lactamase-positive strains (23.5% ) in Group 1 and just 2 beta-lactamase-positive strains (2.85%) in Group 2. One explanation for this finding is the higher consumption of antimicrobial agents in Group 1 children.

The strains from Group 1 children (total 51 isolates) were resistant to rifampicin 15.6% (8  strains, MIC > 8 mg/L), ampicillin 31.37% (16 strains, MIC > 8 mg/L), clarithromycin 21.5% (11 strains, MIC > 16 mg/L), cotrimoxazole 9.08% (5 strains, MIC > 4 mg/L). 

The strains from Group 1 children (total 23 isolates) were resistan to ampicillin 1.42% (1 strain, MIC > 2 mg/L), and there was no resistance to rifampicin.

Further investigations revealed that patients who carried *H. influenzae* suffered from rhinosinusitis for an average of 136 days per year and from otitis media for 92 days per year. Children with tuberculosis but without carriage of *H. influenzae* suffered from otitis media for 14 days per year.

## 4. Conclusion

The carriage of potential bacterial respiratory pathogens in children treated for tuberculosis is underestimated.   The application of molecular epidemiology methods may determine if these organisms are hospital or  community acquired.

## Figures and Tables

**Table 1:** Strains of *H. influenzae* isolated from patients with tuberculosis (Group 1) and children without tuberculosis (Gorup 2).

	No patients	Strains	Patients with one strain	Patients withmultiple isolates
Group 1 (children with tuberculosis)	43	51	26 (60.4%)	17 (39.5%)
Group 2 (children without tuberculosis, control group)	18	23	13 (18.57%)	5 (7.1%)
